# Evidence Inhibition Responds Reactively to the Salience of Distracting Information during Focused Attention

**DOI:** 10.1371/journal.pone.0062809

**Published:** 2013-04-30

**Authors:** Natalie Wyatt, Liana Machado

**Affiliations:** Department of Psychology and Brain Health Research Centre, University of Otago, Dunedin, New Zealand; University of California, Davis, United States of America

## Abstract

Along with target amplification, distractor inhibition is regarded as a major contributor to selective attention. Some theories suggest that the strength of inhibitory processing is proportional to the salience of the distractor (i.e., inhibition reacts to the distractor intensity). Other theories suggest that the strength of inhibitory processing does not depend on the salience of the distractor (i.e., inhibition does not react to the distractor intensity). The present study aimed to elucidate the relationship between the intensity of a distractor and its subsequent inhibition during focused attention. A flanker task with a variable distractor-target stimulus-onset asynchrony (SOA) was used to measure both distractor interference and distractor inhibition. We manipulated the intensity of the distractor in two separate ways, by varying its distance from the target (Experiment 1) and by varying its brightness (Experiment 2). The results indicate that more intense distractors were associated with both increased interference and stronger distractor inhibition. The latter outcome provides novel support for the reactive inhibition hypothesis, which posits that inhibition reacts to the strength of distractor input, such that more salient distractors elicit stronger inhibition.

## Introduction

Despite the fact that attentional processes have been studied for more than a century [1], much remains unknown about how the human brain selects only the most relevant stimuli for extensive processing. One widely-held view is that our attentional system comprises two mechanisms: target amplification and distractor inhibition [2]–[6]. The first of these mechanisms, target amplification, facilitates and strengthens the neural activity associated with relevant stimuli [7]–[9]. The second, distractor inhibition, reduces the strength of the neural activity associated with irrelevant stimuli [5], [6], [10]–[14]. These mechanisms both function to aid selective attention, yet they are distinct and work independently of each other [15]–[19].

Houghton and Tipper [2], [3] described a model that details the role of both target amplification and distractor inhibition in selective attention. In their model, the processing of a given stimulus depends on whether that stimulus is deemed a target or a distractor in the context of the task at hand. During each task, a template of the target stimulus is encoded with the features that are most relevant. When a stimulus is perceived, its features are compared to the features of the target template. If the features of the current stimulus match the target template, then processing of that stimulus is amplified. If the features of the current stimulus do not match the target template, then processing of that stimulus is inhibited. Thus, this dual-mechanism system results in differential processing of target and distractor stimuli, characterised by enhancement of target stimuli and suppression of distractor stimuli.

Houghton and Tipper’s [2], [3] model further explains that the processing strength of both target and distractor stimuli is determined by the intensity of the incoming stimulus. To illustrate, detection of a stimulus activates the selective attention process, and then the strength of the processing that ensues is proportional to the intensity of the activating stimulus. As such, an intense target stimulus will be amplified more strongly than a weak target stimulus, and an intense distractor stimulus will be inhibited more strongly than a weak distractor stimulus. Thus, a key implication of this component of their model is that target amplification is applied in proportion to the strength of the target, and distractor inhibition is applied in proportion to the strength of the distractor. The latter premise will be referred to here as the reactive inhibition hypothesis.

Research exploring the relationship between the strength of a given distractor and the strength of distractor inhibition has provided supportive evidence that inhibition is applied reactively, such that stronger distractors trigger stronger inhibition [20]–[23]. In addition to these studies investigating inhibition in the context of supraliminal distractors, support for the reactive inhibition hypothesis has been reported in the context of subliminal distractors [24]. One obvious benefit of a reactive system over a non-reactive system is that inhibition can respond flexibly to varied distractors, providing higher levels of suppression as needed, thereby assisting toward optimizing the accuracy and speed of responses to targets [20].

Further evidence that the strength of distractor inhibition depends on the intensity of the distractor comes from Schlaghecken and Eimer [25]. Using a flanker task in which they manipulated the strength of the distractor by masking it after a variable interval, these authors found a significant inhibitory effect when there was a long delay between the distractor and a subsequent mask, but not when there was a short delay. This indicated that inhibition was stronger when there was sufficient time for the distractor stimulus to be more thoroughly processed prior to mask onset. Furthermore, an inhibitory effect did not emerge when the distractor was made weaker by a degradation filter. These results were interpreted as indicating that the distractor had to be strongly processed in order to trigger inhibition. Schlaghecken and Eimer concluded that the strength of the distractor must meet a certain threshold before inhibition will be applied, which makes sense from the perspective that weak distracting information should not necessitate inhibition in order to prevent elicitation of a behavioural response. The suggestion that distractors must elicit a strong neural response in order to trigger inhibitory processing is consistent, at least in a general sense, with distractor inhibition behaving reactively.

Although a number of studies have provided data that are supportive of inhibition responding reactively to the strength of distractor input, the existing literature is not unanimous on this viewpoint [26]. Some researchers have instead proposed that the strength of distractor input does not influence the strength of inhibitory processing [27]–[29], and thus stronger distractor input influences inhibitory effects in the exact opposite manner to that proposed by the reactive inhibition hypothesis. Given that the amount of inhibition applied must overwhelm the excitatory processing elicited by the distractor stimulus in order for the net processing to produce an observable inhibitory effect, if inhibition does not respond reactively to stronger distractor input, then the increase in excitatory processing should overwhelm inhibitory processing, thereby preventing inhibitory effects.

A core aspect of this second view is that the strength of inhibition applied to a distractor does not depend on the intensity of the distractor. This will be referred to here as the non-reactive inhibition hypothesis. Key supportive evidence for this theory comes from Ortells et al. [29], who used a modified negative priming task in which a single word served as the distractor stimulus, followed by a target word that required categorisation. In negative priming studies, responses are typically delayed if the target stimulus matches or is related to the previously viewed distractor stimulus, an effect that is referred to as negative priming and is often used as an index of inhibition [30]. Ortells et al. found that the evidence of distractor inhibition (measured via negative priming) was strongest when the distractor display was brief. They interpreted this result as indicating that only weakly represented distractors led to inhibition. Ortells et al. concluded that distractor inhibition does not strengthen when the distractor representation is particularly intense; instead inhibition remains at the same strength and a significant inhibitory effect measured at the behavioural level emerges only when distractor processing is weak enough to be overcome.

**Figure 1:**
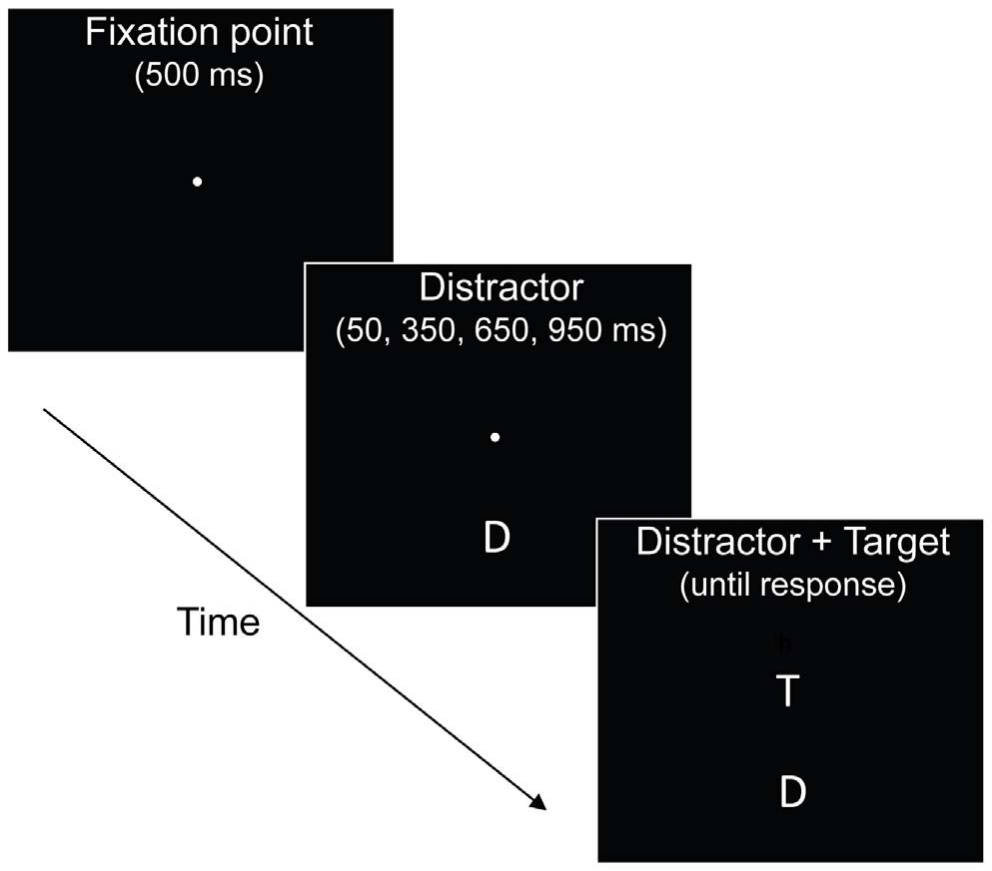
Schematic illustration of the basic paradigm employed, with ‘T’ denoting a target and ‘D’ denoting a distractor (the actual stimulus set comprised a red and a green square in Experiment 1 and two consonants in Experiment 2). A distractor appeared either above or below center at random followed by a central target, which participants identified by pressing one of two buttons. The identities of the distractor and target were either the same (compatible) or different (incompatible). In Experiment 2B, the distractor and target appeared simultaneously.

The present study aimed to determine whether the reactive or non-reactive inhibition hypothesis accurately describes the influence of distractor intensity on the strength of inhibitory processing during focused attention. To yield a detailed characterization of the influences of distractor intensity, we chose a focused attention paradigm that provides measures of both distractor interference and distractor inhibition and also tracks their time course [31]–[35]. The paradigm uses a basic flanker task [36] with a variable temporal interval between distractor and target onset (see Figure 1). During each trial, a distractor appears in the periphery followed by a central target. The identity of the distractor is either compatible or incompatible with the target. When the distractor and the target appear in close temporal proximity, a positive compatibility effect occurs, reflecting faster response latencies when the distractor identity is associated with the target; this provides a measure of distractor interference, which is maximal at the short distractor-target stimulus onset asynchrony (SOA). As the distractor-target SOA lengthens, the positive compatibility effect diminishes and by the long SOA it typically reverses into a negative compatibility effect, reflecting slower response latencies when the distractor identity is associated with the target; this provides a measure of distractor inhibition (regarding the distinction between interference effects and inhibitory effects, see [37]).

Past research supports the utility of this paradigm for assessing the strength of distractor inhibition. For example, it has been shown that the negative compatibility effect that arises at longer distractor-target delays reflects slowing on compatible trials and not speeding on incompatible trials and further that it does not depend on removal of the distractor, all of which are consistent with inhibition of the distracting information causing the negative compatibility effect [35]. Further indication that distractor inhibition underlies the negative compatibility effect comes from a study demonstrating that older adults and patients with Parkinson’s disease do not show this reversal of the compatibility effect and instead show amplified positive compatibility effects [31], which converges with the large body of literature supporting age- and PD-related inhibitory deficits [30], [38]. All of the studies to date utilizing the current paradigm have demonstrated that the short distractor-target SOA always yields a positive compatibility effect and that negative compatibility effects do not arise until longer SOAs (see in particular [34]), which is consistent with claims stemming from other paradigms that it takes time for inhibitory effects to manifest at a behavioural level as inhibition mounts over time and must overcome the excitation elicited by distractor onset [3], [26].

The main advantage of the current paradigm for assessing the relationship between distractor salience and inhibitory processing is that it tracks the effects of the distractor over time, thus providing a more detailed indication of how increases in distractor salience influence distractor processing in terms of both initial distraction and subsequent inhibitory processing. This paradigm has been used previously to assess another aspect of Houghton and Tipper’s [2], [3] model–the dependence of inhibitory effects on removal of the distractor [35]; however, that study did not address the reactive inhibition property of the model. The current study marks the first to utilize this paradigm to test the reactive inhibition hypothesis.

To provide converging evidence as to whether inhibition reacts to distractor salience, we manipulated the intensity of the distractor using two different methods. In Experiment 1, we varied the proximity of the distractor to the target. In Experiment 2, we varied the brightness of the distractor compared to the target. Both of these methods have been shown to influence how strongly the distractor interferes with responses to the target [39]–[51]. In the current paradigm, stronger distractor interference should cause an increase in the positive compatibility effect measured at the short distractor-target SOA. The key question here is whether inhibition will respond reactively to increases in distractor salience. If the reactive inhibition hypothesis put forward by Houghton and Tipper [2], [3] accurately describes the influence of distractor intensity on the strength of inhibitory processing, then more intense distractors (either closer to the target or brighter than the target) should elicit stronger distractor inhibition (as measured by the size of the negative compatibility effect at the long distractor-target SOA). On the other hand, if inhibition does not respond reactively to the strength of distractor input, then more intense distractors should not elicit stronger inhibition and thus weaker negative compatibility effects should result due to the stronger excitatory input overwhelming inhibitory processing.

## Experiment 1: Distractor Proximity

This first experiment explored whether the proximity of the distractor stimulus determines the strength of distractor inhibition. Previous research indicates that the nearer the distractor appears to the target, the more the distractor interferes with responses to the target [39]–[42], [44], [48], [49], [51], [52]. Based on the reactive inhibition hypothesis put forward by Houghton and Tipper [2], [3], distractors located near to the target should be inhibited more strongly than those located further away. Of the studies cited above, only Fox [41] manipulated the distractor-target separation and measured both interference and inhibition. Intriguingly, Fox’s first experiment provided some support for the reactive inhibition hypothesis, but the second experiment did not (see also [28]). Ultimately, Fox concluded that there is no clear relationship between the strength of interference and inhibition. One factor that may have influenced the lack of a consistent relationship is that the paradigm involved measuring distractor interference within a display but distractor inhibition across two displays (i.e., the inhibitory effect depended on the previous display). With the present paradigm, interference and inhibition are both measured within a display, thus obviating this potential issue.

In this first experiment, the distractor could appear at any one of three possible distances from the target. We expected based on past research that distractors positioned closer to the target would elicit stronger distractor interference (as measured by the positive compatibility effect at the 50 ms distractor-target SOA). The key question under investigation here is whether the intensity of the distractor determines the strength of inhibitory processing, in which case the distractors nearer to the target should also elicit stronger inhibition (as measured by the negative compatibility effect at the 950 ms distractor-target SOA). This outcome would support Houghton and Tipper’s [2], [3] reactive inhibition hypothesis. On the other hand, if inhibition does not respond reactively to distractor intensity [27]–[29], then distractors nearer to the target should overwhelm inhibitory processing and thus lead to a weaker inhibitory effect.

### Methods

#### Participants

Thirty undergraduate students (18–29 years; 19 female; 29 right-handed) participated and were reimbursed at the rate of minimum wage. All participants in each of the experiments reported having normal or corrected-to-normal vision, and no colour-blindness or neurological conditions. This research was approved by the University of Otago Human Ethics Committee and all participants provided written informed consent prior to participation.

#### Stimuli and procedure

The stimuli appeared on a black background. A white fixation dot, subtending approximately 0.3° of visual angle and centred on the screen, appeared at the start of each trial. After 500 ms, a red or green square, which served as the distractor, appeared either above or below the fixation dot. After a variable interval of 50, 350, 650, or 950 ms, a red or green target square appeared at the centre of the screen. The edge-to-edge separation of the distractor and target squares was 1°, 2°, or 3° of visual angle. The position of the distractor (above or below centre) prevented effects of spatial compatibility with the lateralized response [53]. The distractor and target squares each subtended approximately 1° of visual angle and both remained on the screen until the computer detected a response or 1500 ms elapsed from the time that the target appeared. A tone sounded for 500 ms if the participant responded incorrectly, responded in less than 100 ms from the time that the target appeared, or failed to respond within 1500 ms. After each trial, the screen turned black for 2000 ms before the start of the next trial. The condition of distractor-target separation (1°, 2°, or 3°) was blocked, with the order of the blocks counterbalanced across participants. The remaining conditions of distractor location (above or below), distractor colour (red or green), target colour (red or green) and distractor-target SOA (50, 350, 650, or 950 ms) occurred randomly with the constraint that each combination of conditions was presented equally often within blocks of 32 trials. During half of the trials the colours of the distractor and the target matched (compatible trials), and during the other half of the trials the colours of the distractor and the target differed (incompatible trials).

Participants sat approximately 57 cm from the monitor in a dimly illuminated room. The experimenter instructed the participants to maintain fixation on the centre of the screen, and to respond as fast as they accurately can to the colour of the square that appears at the centre of the screen by pressing one of the two buttons on the keypad of a gravis joystick using the index and middle fingers of their dominant hand. The buttons measured 2 cm in diameter and had an edge-to-edge separation of 2 cm. The left button indicated red and the right button indicated green for half of the participants, and the opposite stimulus-response mapping was assigned to the other half of the participants. For each level of distractor-target separation, participants completed 32 practice trials followed by 160 experimental trials.

### Results

**Figure 2:**
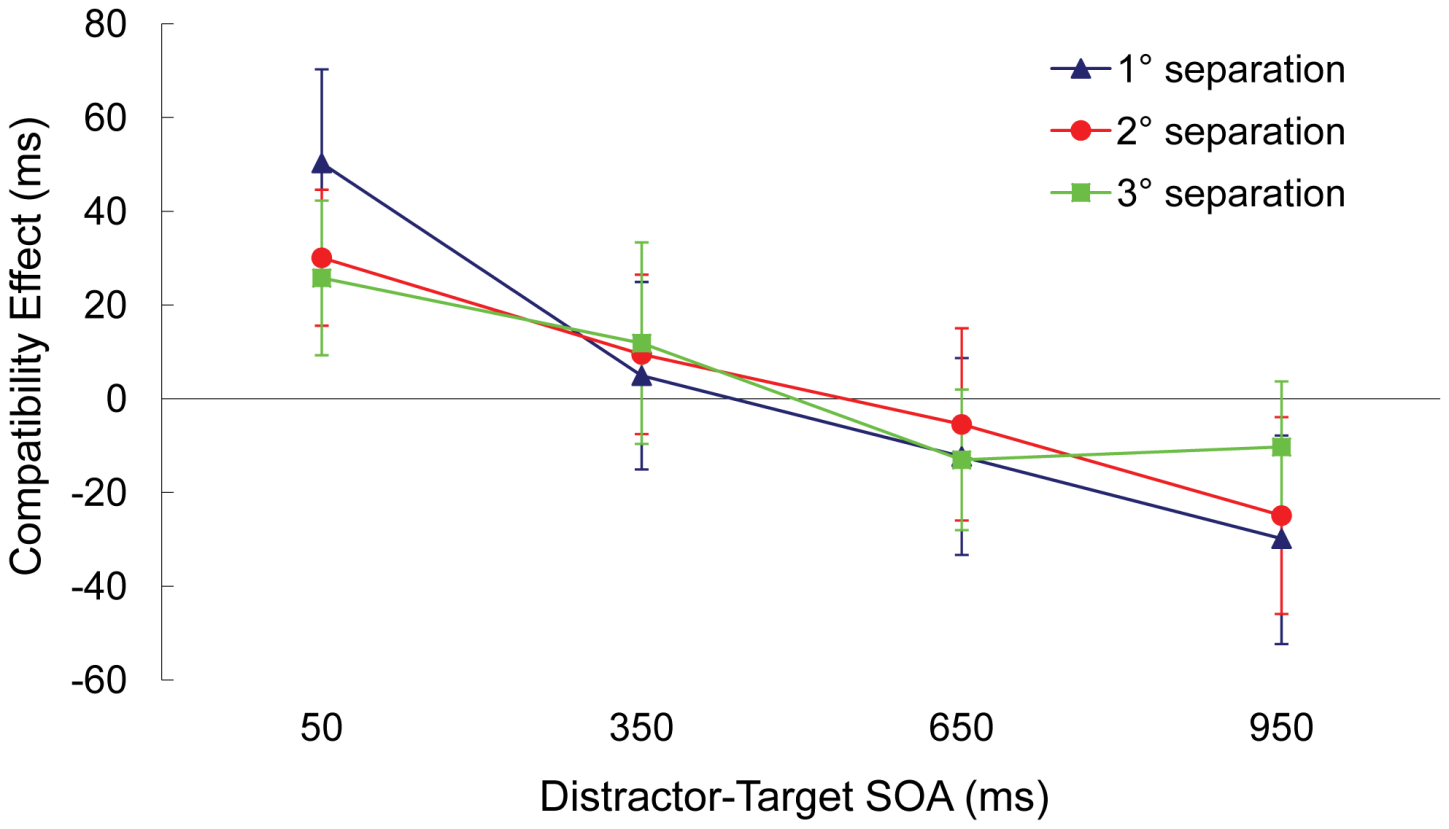
Experiment 1: For each level of distractor-target separation (1°, 2°, or 3°), the size of the compatibility effect in milliseconds for each distractor-target SOA. The compatibility effect equals response latencies on incompatible minus compatible trials. Error bars represent standard deviation.

Table 1 and Figure 2 summarize the results. Only 1.7% of responses were incorrect. Error rates were submitted to an ANOVA with distractor-target separation (1°, 2°, or 3°), distractor-target SOA (50, 350, 650, or 950 ms) and compatibility (compatible or incompatible) as within-subjects variables. The results showed a main effect of SOA, *F*(3, 87) = 5.666, *p* = .002, indicating that the frequency of errors decreased as the SOA lengthened. No other manipulated variables influenced error rates (*p*>.05 in all cases), and in particular the distractor-target separation variable did not influence error rates (*p*>.5 in all cases).

**Table 1:** Experiment 1: Means and Standard Deviations of the Median Reaction Times (in Milliseconds) for Correct Responses and Mean Percentage of Incorrect Responses for Each Condition.

		SOA												
		50			350			650			950			
Distractorproximity	Compatibility	*M*	*SD*	*%*	*M*	*SD*	*%*	*M*	*SD*	*%*	*M*	*SD*	*%*	
**1°**	**Incompatible**	535	99	3.8	459	82	1.7	440	89	1.3	430	87	1.2	
	**Compatible**	485	87	1.7	454	87	2.0	452	85	1.8	459	97	1.0	
**2°**	**Incompatible**	522	108	2.7	461	101	0.8	443	104	1.2	435	97	1.5	
	**Compatible**	492	97	1.8	451	96	2.3	449	99	2.2	461	107	0.8	
**3°**	**Incompatible**	485	83	2.5	457	88	1.7	437	91	2.2	436	100	1.2	
	**Compatible**	511	88	2.0	445	84	1.5	450	94	1.8	446	96	1.0	

Median reaction times for correct responses were submitted to an ANOVA with distractor-target separation (1°, 2°, or 3°), distractor-target SOA (50, 350, 650, or 950 ms) and compatibility (compatible or incompatible) as within-subjects variables. The results showed that reaction times decreased as the SOA lengthened, *F*(3, 87) = 86.022, *p*<.001. No main effect of compatibility emerged, *F*(1, 29) = 0. 808, *p* = .620, but SOA and compatibility interacted, *F*(3, 87) = 39.626, *p*<.001. In addition, there was a three-way interaction of distractor-target separation, SOA and compatibility, *F*(6, 174) = 2.697, *p* = .016. No other effects of the distractor-target separation were reliable (*p*>.5).

Separate ANOVAs for each SOA compared compatibility for the nearest and furthest distractor trials. The compatibility effect was significantly more positive for near distractors compared to the furthest distractors at the 50 ms SOA (+50 ms vs. +26 ms), *F*(1, 29) = 5.197, *p* = .028, which indicates that near distractors produced more interference. Of particular relevance here, the negative compatibility effect was significantly more negative for near distractors compared to the furthest distractors at the 950 ms SOA (−29 ms vs. −10 ms), *F*(1, 29) = 5.835, *p* = .021, which indicates that more salient distractors produced stronger inhibition. Reliable differences between the compatibility effects for the nearest and the furthest distractors did not emerge at the other SOAs (*p*>.6).

### Discussion

To clarify the relationship between the intensity of distractor stimuli and subsequent inhibitory processing, in particular whether distractor inhibition behaves reactively such that more intense distractors lead to stronger development of distractor inhibition, in the current experiment we manipulated distractor intensity by varying the proximity of the distractor to the target. As expected, we found a larger positive compatibility effect at the 50 ms SOA for the distractors nearest to the target, compared to distractors furthest from the target. This indicates that interference increased when the distractor appeared close to the target, replicating earlier findings [39]–[42], [44], [48], [49], [51], [52]. Furthermore, as predicted by Houghton and Tipper’s [2], [3] reactive inhibition hypothesis, a larger negative compatibility effect emerged at the 950 ms SOA for the nearest distractors, compared to the furthest distractors. This evidence of stronger inhibition for distractors nearer to the target goes against the non-reactive inhibition hypothesis, which predicts that inhibition should not strengthen in proportion to the intensity of the distractor and thus inhibitory effects should be less evident for stronger distractors.

## Experiment 2: Distractor Luminance

Experiment 2 investigated how the brightness of the distractor stimulus affects the strength of distractor inhibition. Previous research has shown that the larger the contrast between the distractor and the background, the more the distractor interferes with responses to the target [43], [45], [46], [54]. Thus, here we adjusted the intensity of the distractor by manipulating its luminance. This approach required a departure from the coloured squares employed in the previous experiment. While colour is generally categorised based on its hue (e.g., red, blue, green), changing the luminance of a hue can alter interpretation of the colour [55], [56]. We overcame this obstacle by requiring the participants to respond to the identity of grey letters, rather than to the colour of squares. This allowed the brightness of the stimuli to be manipulated without altering their identity. Furthermore, as degrading the target stimuli can affect the strength of distractor inhibition [57]–[59], the luminance of the target letters was held constant across the different levels of distractor luminance.

To test whether reactive or non-reactive models more accurately describe the influence of distractor luminance on the strength of distractor inhibition, we implemented two levels of distractor luminance: brighter or dimmer than the target. Consistent with past research [43], [45], [46], [54], we expected brighter distractors to elicit stronger interference effects (as measured by the positive compatibility effect at the short distractor-target SOA). If distractor inhibition is applied in proportion to the strength of distractor input, then brighter distractors should also elicit stronger distractor inhibition (as measured by the negative compatibility effect at the long distractor-target SOA). This outcome would support Houghton and Tipper’s [2], [3] reactive inhibition hypothesis. On the other hand, if distractor inhibition does not respond reactively to the strength of distractor input, then brighter distractors should elicit weaker inhibitory effects due to the stronger excitatory input counteracting the effects of inhibition.

## Experiment 2A

### Methods

#### Participants

Thirty undergraduate psychology students (19–32 years; 19 female; 26 right-handed) participated in association with a course.

#### Stimuli and procedure

The stimuli and procedure were similar to Experiment 1 except that letters served as the distractor and target stimuli, the separation between the distractor and target was fixed at 2°, and the luminance of the distractor varied. Two consonants (V and Z, N and S, or B and X) served as the possible distractor and target stimuli for each participant and the stimulus-response mapping was counterbalanced across participants. The distractor and target letters each subtended approximately 1° of visual angle in height. The target was always mid grey (RGB value = [153 153 153]). The distractor was either light grey (RGB value = [229 229 229]) and therefore brighter than the target, or dark grey (RGB value = [77 77 77]) and therefore dimmer than the target. The stimuli appeared on a black background. Furthermore, the stimuli were presented via MATLAB and the Psychophysics Toolbox [60], [61].

The condition of distractor luminance (brighter or dimmer than the target) was blocked, and the order of the blocks was counterbalanced across participants. Each combination of the remaining conditions of distractor location (above or below), distractor identity (one of the two consonants), target identity (one of the two consonants) and distractor-target SOA (50, 350, 650, or 950 ms) occurred randomly and equally often within blocks of 32 trials.

Each of the two consonants selected for each participant was assigned to a response button, and the stimulus-response mapping was displayed on the screen prior to each block. Using the index and middle fingers of their dominant hand, participants indicated the identity of the central letter by pressing on a standard Microsoft keyboard either the B or M key, each of which was covered by a white circular sticker. For each level of distractor luminance, participants completed 20 practice trials followed by 256 experimental trials.

### Results

**Figure 3:**
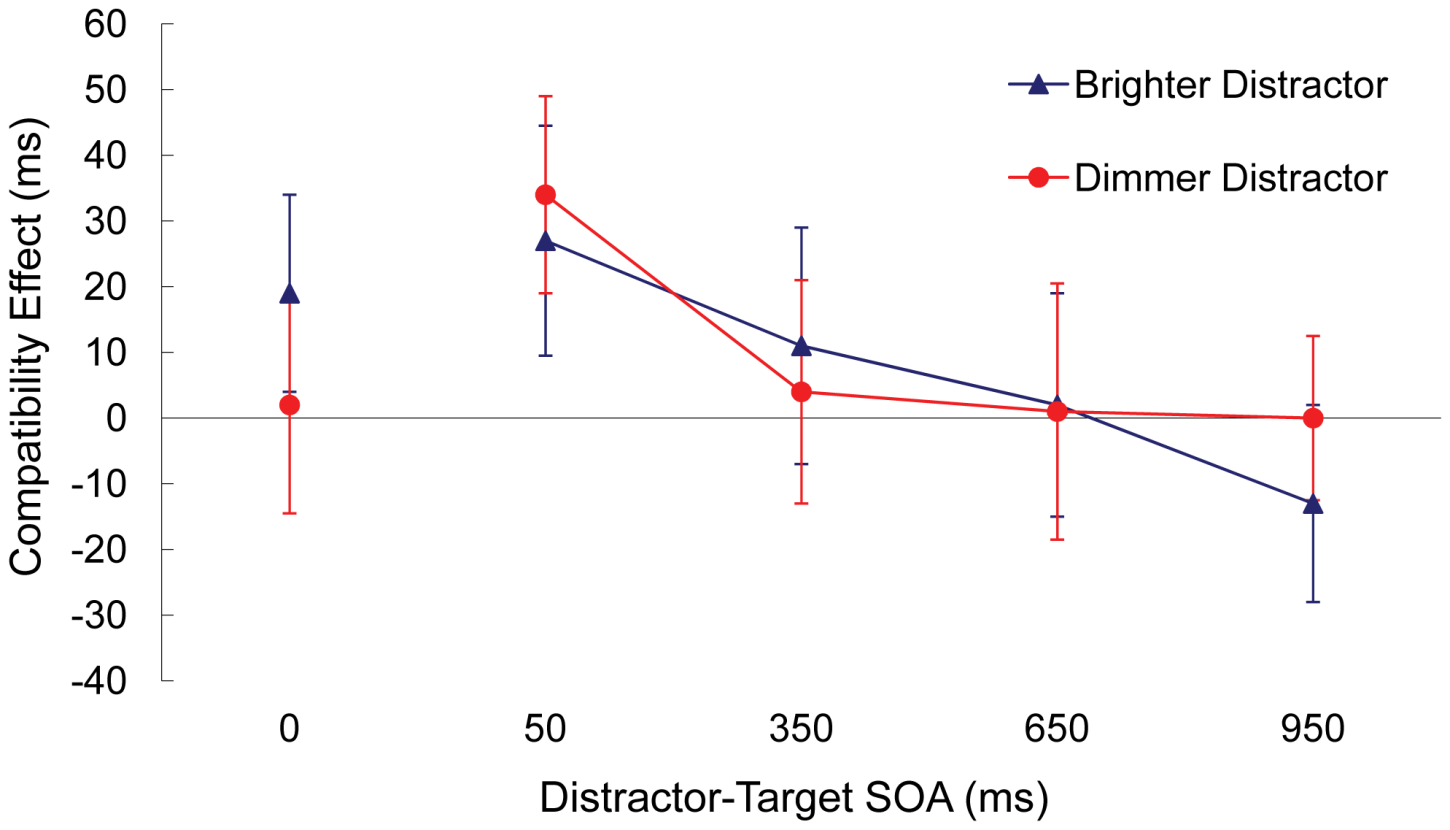
Experiment 2: For each level of distractor luminance (brighter or dimmer than the target), the size of the compatibility effect in milliseconds for each distractor-target SOA (note that the 50, 350, 650, and 950 ms SOAs pertain to Experiment 2A and the 0 ms SOA pertains to Experiment 2B). The compatibility effect equals response latencies on incompatible minus compatible trials. Error bars represent standard deviation.

Table 2 and Figure 3 (see 50, 350, 650, and 950 ms SOAs) summarize the results. Only 3.4% of responses were incorrect. Error rates were submitted to an ANOVA with distractor luminance (brighter or dimmer), distractor-target SOA (50, 350, 650, or 950 ms) and compatibility (compatible or incompatible) as within-subjects variables. The results revealed that the frequency of errors decreased as the SOA lengthened, *F*(3, 87) = 5.545, *p* = .002. Fewer errors occurred on compatible than incompatible trials, *F*(1, 29) = 4.828, *p* = .034. The effect of compatibility depended on the SOA, *F*(3, 87) = 5.806, *p* = .001, reflecting a reduction in the effect of compatibility as the SOA lengthened. In addition, SOA and distractor luminance interacted, *F*(3, 87) = 3.159, *p* = .028, indicating that the effect of SOA was stronger for brighter distractors than dimmer distractors. No other manipulated variables influenced error rates (*p*>.2 in all cases).

**Table 2:** Experiment 2: Means and Standard Deviations of the Median Reaction Times (in Milliseconds) for Correct Responses and Mean Percentage of Incorrect Responses for Each Condition.

		SOA														
		Experiment 2B			Experiment 2A											
		0			50			350			650			950		
Distractor luminance	Compatibility	*M*	*SD*	*%*	*M*	*SD*	*%*	*M*	*SD*	*%*	*M*	*SD*	*%*	*M*	*SD*	*%*
**Brighter**	**Incompatible**	511	66	3.1	552	66	6.4	482	63	2.9	462	56	3.5	454	56	2.5
	**Compatible**	492	64	3.8	525	63	4.2	471	62	3.3	460	59	3.0	467	60	2.0
**Dimmer**	**Incompatible**	494	74	3.9	546	61	5.7	479	53	3.5	460	66	3.0	461	54	3.2
	**Compatible**	492	77	2.5	512	58	1.9	475	66	3.3	459	58	3.2	461	58	3.5

Note that the 50, 350, 650, and 950 ms SOAs pertain to Experiment 2A and the 0 ms SOA pertains to Experiment 2B.

Median reaction times for correct responses were submitted to an ANOVA with distractor luminance (brighter or dimmer), distractor-target SOA (50, 350, 650, or 950 ms) and compatibility (compatible or incompatible) as within-subjects variables. The results revealed that reaction times decreased as SOA increased, *F*(3, 87) = 187.770, *p*<.001. Reaction times were faster on compatible than incompatible trials, *F*(1, 29) = 4.935, *p* = .032. The magnitude of the compatibility effect reduced as SOA lengthened, *F*(3, 87) = 16.891, *p*<.001. No effects of distractor luminance were reliable (*p*>.1), including the three-way interaction of distractor luminance, SOA and compatibility, *F*(3, 87) = 1.554, *p* = .205. Note that testing the reactive inhibition hypothesis does not depend on a three-way interaction [62].

To test the reactive inhibition hypothesis, we needed to determine whether the magnitude of the negative compatibility effect depended on the level of distractor luminance. Based on past studies utilizing the current paradigm, we expected evidence of distractor inhibition to peak at the long SOA. Consistent with the reactive inhibition hypothesis, an a priori ANOVA of the 950 ms SOA revealed that the compatibility effect depended on the luminance of the distractor, *F*(1, 29) = 7.773, *p* = .009, with a significant negative effect arising for brighter distractors (−13 ms), *t*(29) = 2.366, *p* = .024, but not for dimmer distractors (0 ms).

### Discussion

The present experiment aimed to provide further evidence of the relationship between the intensity of a distractor and its subsequent inhibition. The results indicate that brighter distractors elicited a stronger inhibitory effect, which is consistent with the reactive inhibition hypothesis put forward by Houghton and Tipper [2], [3]. However, an obvious limitation of the present data set is that the brightness of the distractors did not affect the strength of interference measured at the short distractor-target SOA. Thus the present results failed to support past reports that more salient distractors produce larger interference effects [45]–[47], [63]–[67]. Given that the shortest distractor-target SOA assessed in the current experiment was 50 ms, it could be the case that the brighter distractors did elicit stronger interference, however the increased interference had already waned by the time of measurement at the shortest distractor-target delay, possibly due to stronger inhibition. This could explain the absence of a difference in interference between the brighter and dimmer distractors in the current experiment, despite past literature indicating that bright distractors should elicit stronger interference effects. The next experiment considered this possibility by presenting the distractor and target simultaneously.

## Experiment 2B

In Experiment 2A, there was no evidence that distractor interference was affected by adjusting the brightness of the distractor. In light of past reports that more salient distractors produce larger interference effects [45]–[47], [63]–[67], we speculated that the brighter distractors did elicit stronger interference; however, it was not measurable within the shortest time frame tested (50 ms). Thus, the present experiment measured distractor interference with a simultaneous onset of the target and the distractor, allowing no time for distractor processing to begin before the target appeared. To prevent confounding simultaneous versus sequential onset of the distractor and the target with SOA, whereby a temporal order cue (respond to the second item) is not provided at the shortest SOA due to the distractor and the target appearing at the same time whereas longer distractor-target SOAs provide such a cue, we opted to include only simultaneous onset trials in the current experiment. With simultaneous onset, we predicted that brighter distractors would elicit a larger positive compatibility effect than dimmer distractors, which would confirm that our manipulation of distractor intensity was effective.

### Methods

#### Participants

Thirty undergraduate psychology students (19–27 years; 25 female; 25 right-handed) participated in association with a course.

#### Stimuli and procedure

The stimuli and procedure were the same in all ways to Experiment 2A except that the distractor and target appeared simultaneously, and given this fixed interval, a variable fixation period (400, 600, 800 or 1000 ms) was introduced in order to prevent participants from being able to predict when the target would appear.

### Results

Table 2 and Figure 3 (see 0 ms SOA) summarize the results. Only 3.3% of responses were incorrect. An ANOVA revealed that none of the manipulated variables influenced error rates (*p*>.08 in all cases).

Median reaction times for correct responses were collapsed over fixation period and submitted to an ANOVA with compatibility (compatible or incompatible) and distractor luminance (brighter or dimmer) as within-subjects variables. The results revealed faster reaction times on compatible than incompatible trials, *F*(1, 29) = 6.704, *p* = .014. There was no main effect of distractor luminance *F*(1, 29) = 1.261, *p* = .270. However, the conditions of distractor luminance and compatibility interacted, *F*(1, 29) = 4.339, *p* = .002, reflecting a larger compatibility effect elicited by brighter than dimmer distractors. Separate *t* tests performed on compatibility for each level of luminance revealed a reliable positive compatibility effect for brighter distractors (+19 ms), *t*(29) = 3.491, *p* = .002, but not for dimmer distractors (+2), *t*(29) = 0.417, *p* = .682.

### Discussion

The present experiment showed that when the target and distractor appeared simultaneously, brighter distractors elicited a larger positive compatibility effect than dimmer distractors. This indicates that our manipulation of distractor luminance did affect the strength of interference. Furthermore, this suggests that the similar strength of interference for brighter and dimmer distractors in Experiment 2A may be due to stronger inhibition of brighter distractors attenuating the interference effect within the time frame of the 50 ms SOA.

Compared to the short distractor-target SOA in the previous experiment, interference appears to have weakened in the present experiment. This pattern is consistent with past research that has shown that the positive compatibility effect strengthens when the distractor precedes the target by a short interval [68], [69]. Furthermore, temporal regularity aids selective attention [70], [71]. A combination of these factors may underlie the seemingly weaker interference in the present experiment. Regardless, the present experiment clearly indicates that the brighter distractors caused more interference than the dimmer distractors, thus replicating past studies [43], [45], [46], [54].

## General Discussion

The present study aimed to determine whether reactive or non-reactive inhibition models accurately describe the relationship between the strength of distractor input and the development of distractor inhibition during focused attention. Each of the experiments involved a variable SOA version of the flanker task in which a peripheral distractor preceded a central target [31]–[35]. We manipulated the intensity of the distractor and measured the strength of both distractor interference and distractor inhibition, as indicated by the positive and negative compatibility effects that emerged over the variable distractor-target SOA. The manipulation of distractor intensity entailed variation in distractor-target separation (Experiment 1) or distractor luminance (Experiment 2), both of which effectively modulated the level of interference incited by the distractor, as indicated by changes in the positive compatibility effect during the short period after distractor onset (note that in the case of distractor luminance this was demonstrated between experiments). Of particular relevance here is that, regardless of whether the distractor was intensified by proximity or brightness, more salient distractors elicited larger negative compatibility effects.

These results suggest that the strength of distractor inhibition depended on the intensity of the distractor. Weak distractor input (which was associated with weak distractor interference) produced little evidence of distractor inhibition, whereas strong distractor input (which was associated with strong distractor interference) produced strong and reliable effects of distractor inhibition. This positive relationship between the strength of a distractor and its subsequent inhibition provides support for Houghton and Tipper’s [2], [3] reactive inhibition hypothesis, which posits that the strength of distractor inhibition is determined by the degree to which the distractor activates the selective attention system. The outcomes reported here extend the findings of previous studies that have supported the reactive inhibition hypothesis [20]–[23], [25] by providing converging evidence from two distinct manipulations of distractor salience (proximity and luminance) in the context of a novel focused attention paradigm that tracked distractor interference and the development of inhibitory effects over time and measured both under similar conditions.

One potential alternative account for the current pattern of data could be that increases in target selection difficulty drove the increases in the inhibitory effects, rather than distractor salience being the key factor. Previous studies have provided evidence that making it more difficult to discriminate the target from the distractor leads to increases in the strength of distractor inhibition [72]. In the current Experiment 1, the manipulation of distractor intensity involved varying the distance of the distractor from the target. Placing the distractor closer to the target could have made target selection more difficult. If so, one would expect to see a main effect of distractor-target separation; however, when the distractor was positioned closer to the target there was no hint of slower response latencies or increased error rates (*p*>.5 in both cases). A similar claim about target selection difficulty increasing with distractor intensity could be made for Experiment 2. Increasing the luminance of the distractor could have made target selection more difficult. However, again, the data did not support this possibility: a main effect of distractor luminance did not arise for response latencies or error rates in Experiment 2A (*p*>.6 in both cases) or in Experiment 2B (*p*>.2 in both cases). Thus, the changes in the strength of the inhibitory effects reported here cannot be accounted for by changes in target selection difficulty as the data do not indicate that target selection difficulty increased with distractor salience.

The reactive behaviour of distractor inhibition contradicts the predictions of non-reactive models [26]–[29], which posit that the strength of distractor inhibition does not depend on the intensity of the distractor, thus the effects of distractor inhibition on behaviour should be less robust for more intense distractors due to inhibition being counteracted by the stronger excitatory processing. One possibility suggested by the present data is that, since in many of the past experiments interference and inhibition were measured at only one point in time (rather than tracking their time course), measurement may not have coincided with the peak of interference and inhibition. For example, in the present study when Experiment 2A is considered alone, there appears to be no relationship between the strength of interference and inhibition. It was only when the target and distractor were presented simultaneously (i.e., 0 ms SOA in Experiment 2B) that evidence of a relationship emerged. This issue may be at the root of why the data from some studies suggest that inhibition does not respond reactively to the strength of the distractor.

The current pattern of data adds insight regarding the duration of salience effects. In a recent study, Donk and Soesman [73] suggested that the augmenting influence of increased salience has only a short-term effect, lasting less than 250 ms. However, our data speak against this claim, given the effects of distractor salience that occurred at the 950 ms SOA. One possible cause of this discrepancy relates to the time frame assessed. Donk and Soesman’s SOAs of up to 483 ms may not have been long enough to detect the effect of salience on inhibitory processing. With the exception of one condition in the present experiments, it took at least 650 ms for the effects of salience on inhibitory processing to emerge. By assessing longer-ranging SOAs, the present study revealed that, rather than salience having no effect at longer SOAs, the augmenting influence of salient distractors on interference measured at short SOAs converts into augmented inhibition at long SOAs.

Interestingly, the evidence of reactive inhibition reported here emerged in the context of the distractor remaining present until response. This goes against the idea that an inhibitory rebound triggered by removing the distractor plays a critical role in the manifestation of inhibitory effects, as proposed by Houghton, Tipper and colleagues [2], [3], [22], or more specifically in the manifestation of reactive inhibition [23]. Past studies utilizing the current paradigm that compared the effects of brief versus persistent distractors found that, in the context of distractors with fixed saliency (i.e., distractor intensity did not vary), removing the distractor did not enhance the negative compatibility effect [32], [35]. Thus it seems that, in the absence of manipulating distractor intensity, inhibitory rebound did not significantly contribute to the negative compatibility effect (for a discussion of this issue see [35]). In the current study, the distractor always remained present until after inhibition was assessed and thus we cannot comment on whether removing the distractor would enhance the reactivity of inhibition to the salience of the distractor; however, the current data do clearly provide novel evidence that inhibition can respond reactively to distractor salience without dependence on removal of the distractor.

Another interesting aspect of the current study is that the evidence of reactive inhibition reported here did not depend on response competition in that the negative effects reflect slowing on trials with a compatible distractor (i.e., the negative effects arise in the absence of response competition) [35]. Past studies reporting evidence of inhibition responding reactively to the strength of distractor activation have suggested that the reactive mechanism functions to reduce response competition [21]. Given that the current demonstration of reactive inhibition arose in the absence of response competition (i.e., on trials with a compatible distractor), and thus response competition was not a driving factor, it seems that reducing response competition is not the only function of this salience-sensitive mechanism.

In summary, tested in the context of a focused attention task, the data in the present study support Houghton and Tipper’s [2], [3] hypothesis that the strength of distractor inhibition depends on the intensity of the distractor. In light of evidence emerging from diverse paradigms that inhibition behaves reactively, for example during task switching paradigms [74], it will be interesting to see through future research whether reactivity represents a general operating principle of inhibitory function.
